# Droplet Digital PCR Is an Improved Alternative Method for High-Quality Enumeration of Viable Probiotic Strains

**DOI:** 10.3389/fmicb.2019.03025

**Published:** 2020-01-22

**Authors:** Sarah J. Z. Hansen, Peipei Tang, Anthony Kiefer, Kevin Galles, Connie Wong, Wesley Morovic

**Affiliations:** DuPont Nutrition & Biosciences, Madison, WI, United States

**Keywords:** probiotic, enumeration, *Bifidobacterium animalis* subsp. *lactis* Bl-04, *Lactobacillus acidophilus* NCFM, *B. animalis* subsp. *lactis* HN019, droplet digital PCR, plate count, flow cytometry

## Abstract

Traditional microbiological enumeration methods have long been employed as the standard evaluation procedure for probiotic microorganisms. These methods are labor intensive, have long-time to results and inherently have a high degree of variability – up to 35%. As clinical probiotic and microbiome science continues to grow and develop, it is increasingly important that researchers thoroughly define and deliver the targeted probiotic dose. Furthermore, to establish high quality commercial products, the same dosage level must be administered to consumers. An ISO method for the use of flow cytometry has been established which does speed up the time to results and reduce variability, but the method has not yet gained widespread adoption across the probiotic industry. This is possibly due to expertise needed to implement and maintain a new testing platform in an established quality system. In this study we compare enumeration using plate counts and flow cytometry to the use of droplet digital PCR (ddPCR), which in addition to giving faster time to results than plate count and less variability than both plate count and flow cytometry, has additional benefits such as strain-specific counts. Use of ddPCR gives the ability to design primers to target deletions and single base pair differences which will allow for strain profiling in microbiome analyses. We demonstrate that ddPCR probiotic enumeration results are positively correlated to both plate count and flow cytometry results and should be considered a viable, next generation enumeration method for the evaluation of probiotics.

## Introduction

For decades, bacteria have been used in fermented food applications and for human health benefits as probiotics (Ouwehand et al., 2000; Heller, 2001). Defined as “live microorganisms, which when administered in adequate amounts, confer a health benefit” (Hill et al., 2014), probiotics in dietary supplements typically include species of *Lactobacillus* and *Bifidobacterium* (Morovic et al., 2016; Lugli et al., 2019). Per the definition, it is crucial for the correct quantities of microbes to be ingested to accurately recapitulate clinical study doses and their associated health benefits (Fenster et al., 2019). Prior to human clinicals, probiotic safety is assured to a certain dose using animal toxicology trials along with associated genomic and *in vitro* data (Mukerji et al., 2016; Morovic et al., 2017). Industrially, probiotic products are quantified throughout the production process (Fenster et al., 2019), which is then factored into the formulation of multi-strain products or as individual offerings (Ouwehand et al., 2018). Finished products must be formulated with overage to account for the inherent cell death over time to still make the required label claim in a variety of environmental conditions and product matrices (Jackson et al., 2019). Altogether, robust methods for quantifying probiotics are paramount to ensure efficacy, safety, and quality.

There are several different methods for quantifying probiotics, each with strengths and weaknesses. The most common method is by plate count enumeration, which has been approved for use by several regulatory bodies (ISO, 2006). Plate count enumeration involves diluting microbial samples and measuring the resulting colony forming units (CFU) from initial dividing cells on defined media. For example, the total *Bifidobacterium* population can be quantified with TOS-mupirocin agar (ISO, 2010), which then can be subtracted from the total lactic acid bacterial count from MRS agar. However, species or strains with similar colony morphology cannot easily be distinguished (Herbel et al., 2013). Furthermore, plates typically need 3–5 days to grow visible colonies and have high reproducibility and repeatability error rates (Jackson et al., 2019). Flow cytometry is an increasingly popular method that rapidly and accurately quantifies samples with various dyes to measure cellular functions such as membrane integrity (ISO, 2015), one of the biggest indicators of cell viability (Kramer et al., 2009). This enables the assessment of non-dividing cells, such as viable but not culturable, which would not result in CFU but could also have a probiotic effect (Ouwehand et al., 2000; Davis, 2014). Like plating, flow cytometry measures phenotypic differences and cannot yet detect small genetic differences such as single nucleotide polymorphisms that could differentiate two strains. Since clinical and safety effects of probiotics are strain-specific (Morovic et al., 2018), testing methods must be able to detect small genetic differences.

Molecular methods, such as next-generation sequencing, provide unparalleled strain resolution, but the cost and technical requirements prohibit routine testing (Morovic et al., 2016; Patro et al., 2016). Quantitative PCR methods present the best opportunities to enumerate probiotics at the strain level (Kramer et al., 2009; Villarreal et al., 2013). Digital PCR employs oligos and enzymes to amplify unique genomic targets in individual reactions, such as wells on a chip or oil droplets. Nucleic acids are distributed into each individual reaction in a random fashion. End-point PCR is performed and positive reactions, which contain at least one copy of the target molecule, exhibit a positive fluorescent signal. The number of positive reactions compared to the number of negative reactions allows the target to be quantified according to its Poisson distribution. This concentration of the single copy target, along with the dilution factor, is used to determine the initial quantity of probiotic bacteria. We previously demonstrated using chip-based digital PCR in combination with viability dyes for absolute enumeration of probiotics at the strain resolution in a matter of hours (Hansen et al., 2018). Here, we improve the method by using droplet digital PCR (ddPCR), which increases sample throughput and decreases standard deviation. We then statistically compare ddPCR to plating and flow cytometry using several commercially available probiotic strains to assess its applicability as an improved method in the probiotic industry.

## Materials and Methods

### Sample Collection

**FIGURE 1:**
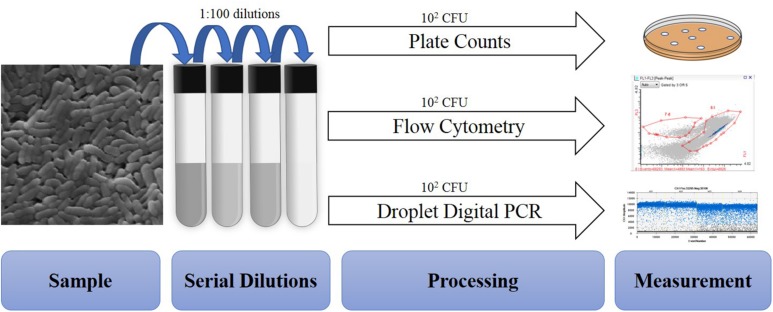
Overview of procedures tested within the study. All samples are similarly diluted prior to different processing and measurement steps.

A total of 50 batches of probiotics including *Lactobacillus acidophilus* NCFM (20), *Bifidobacterium animalis* subsp. *lactis* Bi-07 (9), *Lactobacillus plantarum* Lp-115 (8), *B. animalis* subsp. *lactis* HN019 (8), *B. animalis* subsp. *lactis* Bl-04 (3), and *L. acidophilus* La-14 (2) were collected from a DuPont production site (Madison, WI, United States). Cell quantifications were performed on each batch with four methods: plate count enumeration (Plate), ddPCR (PCR.total), viable ddPCR (PCR.live), and flow cytometry gated for viable cells (Flow.live), dead cells (Flow.dead), and total cells (Flow.total). Replicates for each method were 17 Plate and triplicates for other methods (Figure 1).

### Plate Count Enumeration

**FIGURE 2:**
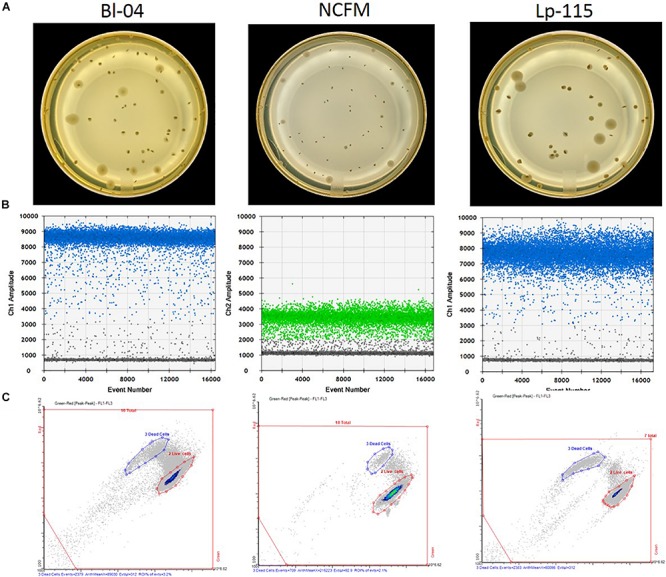
Measurement outputs for the various enumeration technologies. Raw data output per single reactions/colony are shown for **(A)** plate counts, **(B)** ddPCR according to the assay probe FAM (blue) or HEX (green), and **(C)** flow cytometry, for three different probiotic strains. The pictures are examples of typical outputs for each method, which would then be multiplied by the dilution factors to obtain final results.

Freeze-dried probiotic powders were enumerated by standard microbiological pour plating. Briefly, 11 g of freeze-dried probiotic powder were rehydrated in de Man-Rogosa-Sharpe (MRS) broth (p/n 288110; BD, Franklin Lakes, NJ, United States) and homogenized using the Stomacher 400 Circulator (Seward Ltd., Bohemia, NY, United States) before and after a 30 min rehydration period. Rehydrated samples were serially diluted using 0.1% Peptone diluent (p/n FTPW9960; 3M, St. Paul, MN, United States) to an appropriate concentration yielding 25–250 colonies per plate. Plates were inoculated in triplicate with diluted sample and ∼15 ml of MRS agar (p/n 288210; BD, Franklin Lakes, NJ, United States) + 0.05% cysteine-HCl (p/n C7880; Sigma-Aldrich, St. Louis, MO, United States). Only Lp-115 was plated in the absence of cysteine. Plates were incubated at 38°C under anaerobic conditions for 72 h when colonies were enumerated and recorded as viable cell count per gram, considering the dilution factor (Figure 2A).

### Droplet Digital PCR

Freeze dried probiotic powders were analyzed by the ddPCR method outlined previously (Morovic et al., 2018). In short, samples were rehydrated and diluted in Butterfield’s phosphate buffer (p/n R23701; Fisher, Hampton, NH, United States) to an appropriate concentration. When used, 1.2 mL of samples were treated with a specific concentration (Table 1) of viability dye PEMAX (p/n 4900013150; GenIUL, Barcelona, Spain). Treated samples were incubated for 30 min. at 37°C while shaking and protected from light, followed by photoactivation on PMA-Lite device for 15 min. Treated and untreated samples were then bead beaten for specified time (Table 1) in prefilled 2.0-ml tubes containing Triple-Pure high-impact 0.1-mm zirconium beads (D1032-01; Benchmark Scientific, Edison, NJ, United States) in chilled (−20°C) aluminum block with Mini-Beadbeater-96 (p/n 1001; BioSpec Products, Bartlesville, OK, United States). Samples were also processed using the QIAeasy Blood and Tissue kit (p/n 69504, Qiagen, Hilden, Germany) on a QIAcube (Qiagen) using the standard protocol. Each primer and probe assay were designed to target single copy unique sequences within the individual strain genomes (Table 1). Internal and publicly deposited genomes were used as controls for *in silico* testing through the BLAST tool (National Center for Biotechnology Information, Rockville, MD, United States) and showed each to be unique to the strain targeted. The assays for the detection of *B. animalis* subsp. *lactis* Bl-04 and *L. acidophilus* NCFM were previously reported (Hansen et al., 2018). A PCR mixture was created with the primer and probe sequences specified in Table 1. 0.42 μL of PCR grade water, 4.5 μl of forward and reverse primers, 2.08 μl of probe, 12.5 μl of ddPCR Supermix for probes (no dUTP) (p/n 1863024; Bio-Rad, Pleasanton, CA, United States), and 1 μL of bead beaten sample were added to 96 well plates in triplicate (Bio-Rad, Pleasanton, CA, United States). Plates were sealed with pierceable foil (p/n 1814040, 1814000; Bio-Rad), formed into droplets (p/n 1864101; Bio-Rad), then thermocycled (p/n 1851197; Bio-Rad) at 95°C for 10 min, 40 cycles of 95°C for 30 s and Tm°C (see Table 1) for 1 min, and finally 98°C for 10 min with an infinite 10°C hold. Droplets were read and analyzed by QX200^TM^ Droplet Reader and QuantaSoft^TM^ software (Bio-Rad) per manufacturer’s recommendations (Figure 2B). The ddPCR reactions treated with PEMAX are further denoted as PCR.live, and untreated are PCR.total.

**TABLE 1:** Strain-specific primers and probes for NCFM, Bl-04, Bi-07, HN019, Lp-115, and La-14.

	**Primer/Probe name**	**Primer/Probe sequence**	**Tm (°C)**	**Amplicon size (bp)**	**PEMAX conc. (uM)**	**Bead beating time (m)**
NCFM	Laci_ABCins_qF	CCA CGA CCA GAT GTA ACC AA	60	209	3.7	6^a^
	Laci_ABCins_qP	/6-HEX/TAA GCC GAA/ZEN/CAA TGC TGA AAC GAT/3IABkFQ				
	Laci_ABCins_qR	TTA GAA GAT GCC AAC GTC GAG				
Bl-04	Bl04_LcFACoA_qF	CTT CCC AGA AGG CCG GGT	60	98	1.7	15
	Bl04_LcFACoA_qP	/6-FAM/CGA AGA TGA/ZEN/TGT CGG AAC ACA AAC ACC CGG/3IABkFQ				
	Bl04_LcFACoA_qR	CGA GGC CAC GGT GCT CAT ATA GA				
Bi-07	Blac_LfCoAins_qF1	TTC AAG CCG ACG TAC TTG CT	60	178	1.7	15
	Blac_LfCoAins_qP	/5HEX/TC GCC AAT G/ZEN/C CGT CGA CCA T/3IABkFQ/				
	Blac_LfCoAins_qR1	TGA TTC GCA TCA TCG GTC CC				
HN019	Blac_CRISPRdel_qF	TTC GAT GGT TCG CAC AGT GA	60	158	5.0	15
	Blac_CRISPRdel_qP	/56-FAM/AA ACA GGT C/ZEN/A ATC AGC GGC GCA GGG AG/3IABkFQ/				
	Blac_CRISPRdel_qR	GGT CTG ATG CCG CCT GAA AT				
Lp-115	Lp115_CR_RTF	CTT GAT GAC TCT TCT GGG GC	60	165	8.2	15
	Lp115_CR_RTP	/56-FAM/TT GAG TGC A/ZEN/G CGT TGT TTG CGA GCG TCC/3IABkFQ/				
	Lp115_CR_RTR	ACG GGA GTG ATA GAC GTT GAG				
La-14	La14_ABCdel_qF	CCG GTT AAT AAA ATC TTT TCA CCT TG	56	202	3.9	6^a^
	La14_ABCdel_qP	/56-FAM/AG TTG ATC A/ZEN/G TCA GCA AGT AGT GTT ATG G/3IABkFQ/				
	La14_ABCdel_qR	GCA GTT ATT AAT CGT GAT TTG CAT ATA AAT T				

*^*a*^Samples were run for 3 min, rested for 1 min on ice, then run three additional minutes.*

### Flow Cytometry

Freeze dried probiotic powders were analyzed by flow cytometry in accordance with ISO 19344:2015 Protocol B. In short, samples were homogenized and rehydrated like the plate count enumeration method. Samples were further diluted 1:10 in phosphate buffered saline as noted in the ISO method, then treated with Propidium Iodide (p/n P3566; Life Technologies, Carlsbad, CA, United States) and Syto^TM^ 24 (p/n S7559; Life Technologies). Treated samples were incubated at 37°C while shaking and protected from light for 15 min. Samples were analyzed in triplicate on an A-50 Micro flow cytometer instrument (Apogee Flow Systems; Hemel Hempstead, United Kingdom) in accordance with manufacturer’s recommendations. Cytograms were appropriately gated and regions of interest were created to evaluate live, dead and total cell populations (Figure 2C), further denoted Flow.live, Flow.dead, and Flow.total.

### Analysis of Method Agreement

The Pearson correlation coefficient (*r*) of the results obtained from each paired method was calculated, and a linear function was applied to investigate their association. Then the Bland–Altman analysis was conducted to compare the agreement of the two measurements (Bland and Altman, 1986). The relative difference between a pair of measurements [(Method 1 - Method 2)/(average of the two methods)] based on the given two methods was displayed, in relation to the mean of the paired measurements. The overall matching between the two methods was summarized and evaluated by the mean relative difference and the limits of agreement (LOA).

### Analysis of Methods Consistency

The Coefficient of Variation (CV) (also known as the Relative Standard Deviation, RSD) of a given method was calculated based on the average CV of all the 50 batches.

## Results

### Assay Efficiency for ddPCR

**FIGURE 3:**
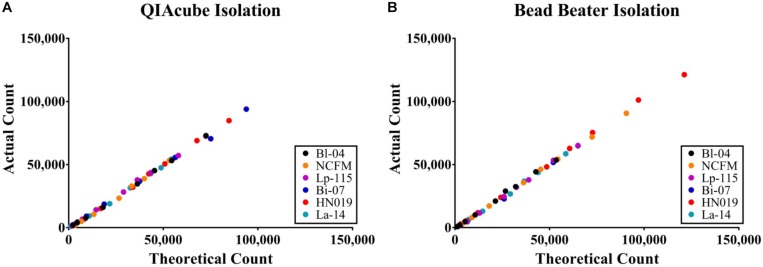
Absolute concentration accuracy of DNA liberation for ddPCR template. The actual count of ddPCR copies compared to the theoretical count were calculated for **(A)** preparations using the QIAeasy blood and tissue kit on a QIAcube and **(B)** bead beating and no subsequent purification steps. Samples were tested in triplicate, but the error bars are too small to visualize.

The efficiency of the PCR assay for each strain was evaluated using both gDNA isolated from the automated kit-based QIAcube system and from our internal DNA liberation method using bead beating on six strains. A dilution series of these DNA samples was created to determine assay efficiency, down to 2.5% of the original sample. Each DNA liberation method yielded similar efficiencies to each other (Table 2), within 5% and were all with an acceptable limit of 90–105% (Bio-Rad reference). When compared to the theoretical yield calculated based on initial ddPCR reading (Figure 3) and dilution calculations, each strain’s *R*^2^ value was over 0.99 demonstrating no difference between our method and the commercial method (Table 2).

**TABLE 2:** Efficiency of DNA liberation methods.

**Strain**	**Bead beating *R*^2^**	**Bead beating efficiency (%)**	**QIAcube *R*^2^**	**QIAcube efficiency (%)**
Bl-04	0.9981	103.11	0.9992	101.05
NCFM	0.9997	100.95	0.9975	103.66
Lp-115	0.9991	102.55	0.9992	100.49
Bi-07	0.9986	102.47	0.9983	98.04
HN019	0.9989	102.31	0.9993	102.03
La-14	0.9982	101.49	0.9981	99.52

### Standard Deviation of the Methods

**FIGURE 4:**
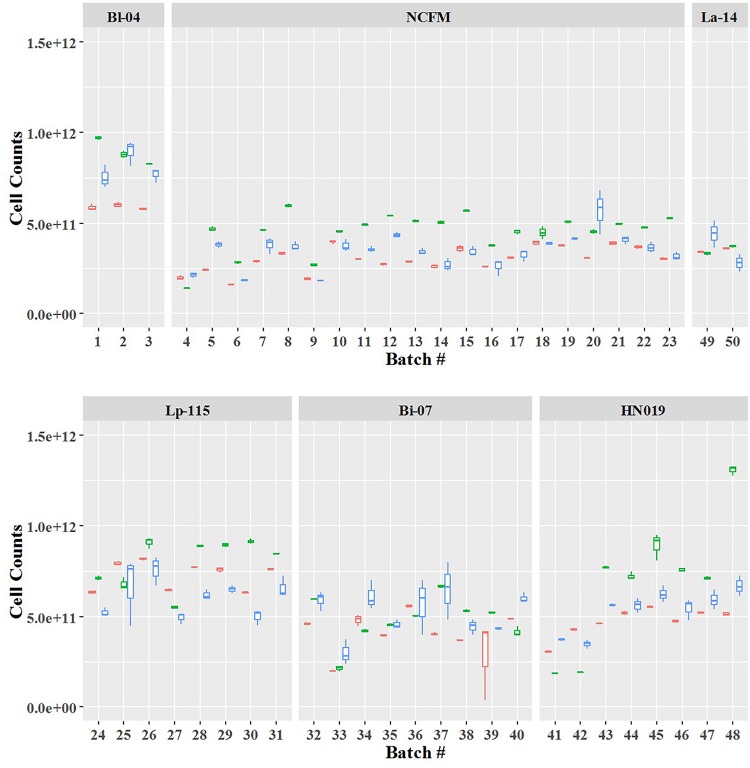
Quantification of six probiotic strains using three different methods. The final counts are shown by batch number. Box plot colors denote the method: red, Flow.live; green, PCR.live; and blue, Plate.

**FIGURE 5:**
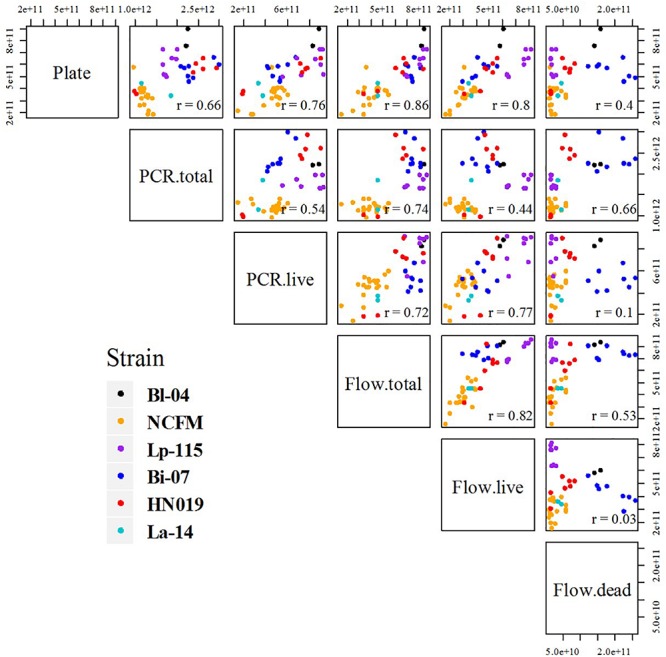
Comparative scatter plot of results between each method and strain. Each plot compares the final results, listing each method combination by row and column. Plots show the results by strain color as mentioned in the legend, and *r* values are noted in the corners.

Cell count measurements of the 50 batches by plate count (Plate), viable ddPCR (PCR.live), and flow cytometry (Flow.live) were displayed in boxplot (Figure 4 and Supplementary Table S1). We focused on the PCR.live and Flow.live methods due to their ability to asses viability and their high correlations with the Plate method as shown in Figure 5. In most cases, the results obtained from the three methods were very close to each other in a practical sense. PCR.live measurements tended to be slightly larger than the other two methods, while Plate measurements exhibited the largest variability among the three (CV = 16.2 ± 4.4%, Table 3) followed by Flow.live (CV = 3.3 ± 1.0%) and PCR.live (CV = 2.0 ± 1.0%) (Table 3).

**TABLE 3:** Variability of the methods.

**Strain**	***N***	**Plate (%)**	**ddPCR.total (%)**	**PCR.live (%)**	**Flow.total (%)**	**Flow.live (%)**	**Flow.dead (%)**
All	50	16.2 ± 4.4	2.3 ± 1.0	2.0 ± 1.0	4.9 ± 1.0	3.3 ± 1.0	4.9 ± 1.5
NCFM	20	15.5 ± 3.9	2.1 ± 0.8	2.1 ± 0.9	10.5 ± 1.3	2.5 ± 1.3	3.8 ± 1.3
Non-NCFM	30	16.8 ± 5.2	2.3 ± 1.0	2.1 ± 1.1	1.3 ± 0.7	3.7 ± 0.7	5.7 ± 1.0
Bi-07	9	19.8 ± 7.7	4.7 ± 1.0	2.1 ± 0.8	1.2 ± 0.9	9.7 ± 0.6	1.4 ± 1.1
Lp-115	8	15.3 ± 1.3	2.0 ± 1.0	1.8 ± 1.4	1.1 ± 0.5	1.1 ± 0.5	2.6 ± 2.2
HN019	8	14.8 ± 4.0	1.5 ± 1.1	2.6 ± 0.7	1.1 ± 0.4	1.3 ± 0.3	2.2 ± 1.4

### Method Correlation

Pearson correlation coefficients (*r*) of the results were calculated and the comparative scatter plots were presented in Figure 5. There were strong correlations between Plate and PCR.live, Plate and flow cytometry (both total and live cells), Flow.live and Flow.total, Flow.live and PCR.live, with *r* = 0.76, 0.86, 0.80, 0.82, and 0.77, respectively. These correlation coefficients were either greater than or similar to previous studies which focused on the comparison of methods for cell enumeration (Naghili et al., 2013; Luedtke and Bosilevac, 2015). Conversely, Flow.dead was poorly related to other methods.

**FIGURE 6:**
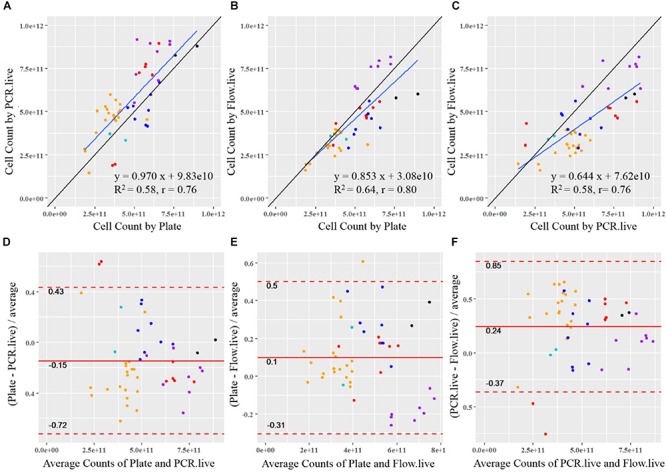
Statistical comparison between the quantitative methods for probiotics. The correlation of results from each sample is shown between **(A)** PCR.live vs. Plate, **(B)** Flow.live vs. Plate, and **(C)** Flow.live vs. PCR.live. The black lines show the theoretical correlation and the blue lines show the trendlines of measurements. Colored dots represent batches by strain with colors identical to Figure 4: black, Bl-04; orange, NCFM; purple, Lp-115; dark blue, Bi-07; red, HN019; light blue, La-14. The agreement between methods is analyzed by Bland–Altman plots between **(D)** PCR.live vs. Plate, **(E)** Flow.live vs. Plate, and **(F)** Flow.live vs. PCR.live.

Cell count results obtained by the three methods were plotted against each other, and a linear function was applied to determine the association between the given two methods (Figures 6A–C). A reference line (*y* = *x*, showing the expected association) was included in each plot to visualize the methods’ agreement. If data points are strictly distributed along the reference line, then the measurements from the two methods are very alike. Plate measurement exhibited strongly positive associations with PCR.live and Flow.live since most of the data spread randomly around the *y* = *x* reference line, with slopes close to 1 (0.970 and 0.853, respectively). The regression line of Plate vs. PCR.live was above the *y* = *x* reference line, in agreement with the previous boxplot in which PCR.live overestimated cell counts compared to Plate results. There was also a strong correlation between the results obtained from PCR.live and Flow.live (*r* = 0.76, slope = 0.644), while the PCR.live results tended to overrate when compared to Flow.live over the high range of measurement values.

### Method Agreement

Bland–Altman analysis was conducted to compare the agreement of cell count measurements among the three methods (Figures 6D–F). The relative difference between a pair of measurements based on the given two methods was displayed, in relation to the mean of the pair of measurements. The values within the LOA summarized the overall matching between the two methods. They are considered similar if the differences are small (close to zero) and are consistent over the range of measure values with a narrow LOA. As shown in Figures 6D,E, the mean relative differences of Plate vs. PCR.live, and Plate vs. Flow.live were −15 and 10%, with 95% LOA of −72 to 0.43% and −31 to 50%, respectively. Based on the results, we would expect an average of 15 and 10% relative differences between Plate vs. PCR.live, and Plate vs. Flow.live, respectively. In addition, 95% of the relative differences of the methods were expected to be within (−72%, 43%) and (−31%, 50%), respectively. Similarly, there was a 24% average relative difference (Figure 6F) between PCR.live and Flow.live, with LOA between −37 and 85%.

## Discussion

With more probiotic manufacturers producing new strains and products, the availability of high-quality, quantitative information is essential to ensure consumer trust in the industry (Jackson et al., 2019). Total cell count is a key metric that is displayed on product labels, along with strain identification. Enumerating products is time consuming and labor intensive, so alternative methods to traditional plate count enumeration need to be evaluated. This research compares three different methods, two of which, flow cytometry and plate count enumeration, are already established methods. We hypothesized that ddPCR is a comparable enumeration method to the gold standard of plate counts, but has additional benefits, including strain specificity, time to results, and high precision. The technical simplicity and benefits of rapid, specific quantification outweigh initial capital investments and makes ddPCR a cost-efficient option for high-throughput, routine analyses. To compare these methods, we analyzed multiple batches of each strain to create a robust matrix of data.

Previous research on plate count enumeration showed an average CV of 15%, which agrees with our findings (Table 3), however, ISO acceptable levels can vary between 15 and 35%. Although the ISO method for flow cytometry reported high variation, literature reports from 3 to 7% CV (Wilkinson, 2018) similar to our results. Overall, ddPCR had a CV of 2.0 ± 1.0% calculated in scientific notation, as opposed to reporting in logarithmic form which lowers the overall CV. Because probiotic products are labeled in CFU in scientific form, all our analysis is done on actual data, not log transformed data. To assess the association between two given methods, Pearson Correlation Coefficient (*r*) was calculated and a linear function was applied. ddPCR exhibited a highly positive correlation with plate count enumeration (*r* = 0.76) and is higher than previously reported for acceptable equivalent methods (Mukaka, 2012; Naghili et al., 2013; Luedtke and Bosilevac, 2015). Similar equivalence was observed between flow cytometry verses plate count enumeration or ddPCR with an *r* = 0.80 and *r* = 0.77, respectively. The slope of the linear regression of plating compared to ddPCR or flow cytometry was 0.970 and 0.853, respectively. Therefore, flow cytometry was less consistent over the data range and tended to underrate across the high range of measurement values. Based on Brand–Altman analysis, we would expect an average of 15% relative difference between plating and ddPCR, but further optimization or new viability dyes may decrease this difference.

The standard definition of a probiotic includes “live cells,” but the term “live cells” is not well defined. For example, Pegg (1989) call viability “the ability of a treated sample to exhibit a specific function or functions, expressed as a proportion of the same function exhibited by the same samples before treatment or an identical fresh untreated sample.” The probiotic industry has interpreted the definition of live cells to be CFU on an agar medium, which has been used for clinical research and production quality assessment. However, this does not account for postbiotics (dead cells that still have a health benefit) or viable but not culturable cells. The PEMAX dye used in this report has a dual mechanism for determining viability: cell membrane integrity and active metabolism to maintain homeostasis (Codony et al., 2015). Dye concentrations and assay parameters were optimized to target plate count enumeration due to its long history of use as a probiotic industry standard. Future dyes that provide better or different mechanisms could be adapted for ddPCR assays to better associate with clinical results.

In clinical trials, safety and structure-function claims are based on concentrations of individual or multiple strains in investigational products. Additionally, clinical benefits are associated with specific strains, because small genomic differences can have drastic phenotypic differences (Briczinski et al., 2009; Morovic et al., 2018). For commercial products to claim similar benefits, they must contain the exact strain and concentration throughout shelf life (Jackson et al., 2019). While all three methods listed herein can measure viable concentrations, ddPCR can easily be designed to target deletions and single base pair differences, even in multi-strain products. This is also essential for detecting strains in microbiome datasets. Furthermore, as molecular mechanisms for probiotic effects become better defined, ddPCR can be adapted with different technologies to measure gene expression levels to estimate enzyme production. Although we only tested freeze-dried material in this study, ddPCR has the potential to be used with probiotics in other product matrices such as dietary supplements and food. In sum, ddPCR offers a robust method to quantify specific genomic targets.

The probiotic industry has relied on clinical trials to establish health benefits and safety. As molecular tools improve and insights into mode-of-action increase, so do the questions. Here, we show that ddPCR can measure viable probiotics in agreement with current methods like plating and flow cytometry, but its ability to target genetic elements allows for adaption with evolving molecular insights. Future research into specific gene targets and application with shelf-life stability will improve the method. By utilizing precise genetic enumeration methods like ddPCR, the probiotic industry will improve the product quality for consumers.

## Data Availability Statement

All datasets generated for this study are included in the article/Supplementary Material.

## Author Contributions

SH and WM managed ideation and experimental design. WM, AK, and KG optimized assay design and analyzed the results. PT performed statistical analysis. CW was the project manager and contributed to the ideation of the experimental design. All authors contributed to writing and editing the manuscript.

## Conflict of Interest

The authors declare that this study received funding from DuPont Nutrition & Biosciences (DuPont N&B). The funder had the following involvement in the study: all authors are employees of DuPont N&B, the microbial strains used are commercially sold by DuPont N&B, and DuPont N&B reviewed the manuscript prior to submission.

## Supplementary Material

TABLE S1 Raw data of cell quantification results using plate, PCR.live, and Flow.live.
